# Data supporting metabolite profiles of essential oils and SSR molecular markers in *Juniperus rigida* Sieb. et Zucc. from different regions: A potential source of raw materials for the perfume and healthy products

**DOI:** 10.1016/j.dib.2019.104113

**Published:** 2019-06-06

**Authors:** Zehua Liu, Shun Kuang, Mingliang Qing, Dongmei Wang, Dengwu Li

**Affiliations:** College of Forestry, Northwest A&F University, Yangling, Shaanxi 712100, China

**Keywords:** *Juniperus rigida*, Essential oils, GC-MS fingerprint, Principal component analysis

## Abstract

The data presented in this article afford insight into how high-quality origins were basically evaluated viewed from yields of essential oils and how GC-MS fingerprint constructed and analyzed as supplementary materials supporting the results displayed in the article of metabolite profiles of essential oils and SSR molecular markers in *Juniperus rigida* Sieb. et Zucc. from different regions: A potential source of raw materials for the perfume and healthy products Liu et al., 2019. The presented data demonstrate the supplementary instruction of the GC-MS fingerprint analysis results of *Juniperus rigida* from different origins Meng et al., 2016. The data of essential oils yields, similarities and correlation coefficients of GC-MS fingerprint and principal component analysis (PCA) supported the results of high-quality *J. rigida* provenance selection.

**Table:** Specifications table

Subject area	*Agricultural and Biological Sciences*
More specific subject area	*Plant Science*
Type of data	*Table, figure*
How data was acquired	*The similarities, correlation coefficients and principal components analysis of GC-MS chromatograms of J. rigida were investigated using SPSS Package 21.* *The yields of essential oils of J. rigida were draw by Sigma plot 12.0.*
Data format	*Analyzed, raw*
Experimental factors	*The essential oils of J. rigida was isolated by supercritical CO*_*2*_*fluid extraction technology used by**[2]**.*
Experimental features	*The components of J. rigida essential oils were identified by GC-MS and the fingerprint chromatography were collected and analyzed by chemometrics methods.*
Data source location	*Northwest A&F University, Shaanxi, China*
Data accessibility	*Data are available with this article*
Related research article	[1] Z. Liu, S. Kuang, M. Qing, D. Wang, D. Li. Metabolite profiles of essential oils and SSR molecular markers in *Juniperus rigida* Sieb. et Zucc. from different regions: A potential source of raw materials for the perfume and healthy products. J. Ind. Crop. Prod., 133, 2019, 424.

**Table:** 

**Value of the data** • The data of essential oil yields provided basic information for screening *J. rigida* which contains enrich essential oils, the data can also be used for displays as windows for screening high-quality J. rigida provenance. • The data of similarities and correlation coefficients of chromatograms appeared characteristic of the specific essential oils, supporting disparities among plants derived from different regions. This data helped with the classification of the chromatography of GC-MS fingerprint into different groups and provided auxiliary information for quality evaluation of *J. rigida* provenances. • The principal component analysis (PCA) of different *J. rigida* origins confirmed the results in hierarchical clustering analysis. Chromatograms were similar within a particular group while significantly distinct between groups. The first two principal components contained the most information of all variables, accounting more than 85% of total variability. These data helped with high-quality *J. rigida* provenance selection and were in supplementary materials for the indicating components screen.

## 1 Data

**Fig. 1:**
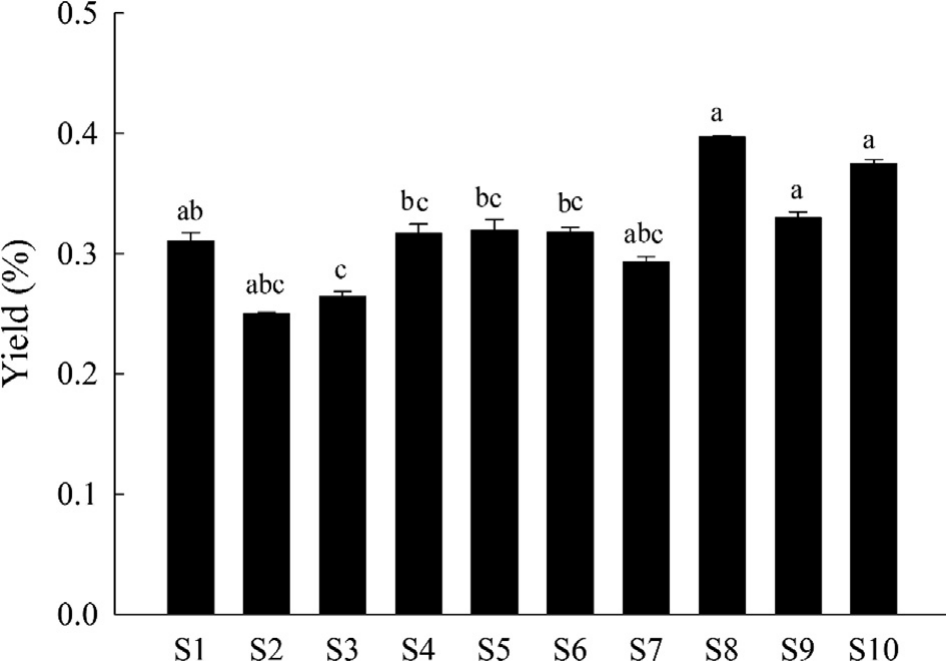
Yields of essential oils of *J. rigida* samples in different regions.

**Fig. 2:**
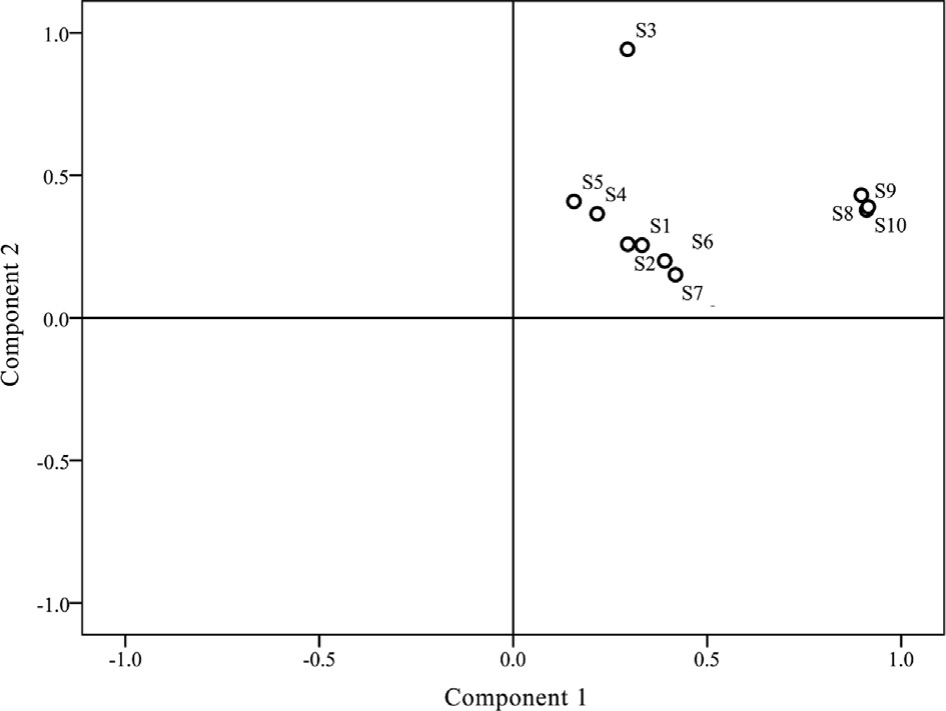
The scores plot generated from principal component analysis (PCA) of variables (S1-10).

Data presented in this article displays a drawing showing the supplementary information for the GC-MS fingerprint analysis. The yields of essential oils of *J. rigida* in different origins were displayed in Fig. 1. The similarities comparison of GC-MS chromatography of *J. rigida* essential oils from different origins was showed in Table 1. The correlation coefficients among different groups were assessed to support the GC-MS fingerprint analysis as well as quality evaluation of *J. rigida* origins (Table 2). The scores plot generated from principal component analysis (PCA) of *J. rigida* variables (S1-10) supported the results of hierarchical clustering analysis in GC-MS chromatography for screening the high-quality origins (Fig. 2).

**Table 1:** Similarities of the GC-MS chromatograms of *J. rigida* samples based on the correlation.

No.	S1	S2	S3	S4	S5	S6	S7	S8	S9	S10
S1	1.000									
S2	0.901	1.000								
S3	0.833	0.824	1.000							
S4	0.983	0.906	0.903	1.000						
S5	0.921	0.945	0.929	0.950	1.000					
S6	0.987	0.914	0.805	0.960	0.911	1.000				
S7	0.951	0.929	0.761	0.917	0.907	0.976	1.000			
S8	0.935	0.883	0.679	0.878	0.833	0.960	0.955	1.000		
S9	0.892	0.881	0.638	0.832	0.808	0.929	0.929	0.978	1.000	
S10	0.906	0.888	0.646	0.844	0.820	0.938	0.949	0.995	0.982	1.000

**Table 2:** Correlation coefficients between individual chromatograms within a group and the group simulative mean chromatogram, and between the group simulative mean chromatogram.

Group	G1	G2	G3
G1	0.985 ± 0.008^a^(n = 3)	0.658^b^	0.924^b^
G2		1^a^(n = 1)	0.862^b^
G3			0.937 ± 0.029^a^(n = 6)

a Correlation coefficient of individual chromatograms to the simulative mean chromatogram of the corresponding group. Values are the mean ± SD.

b Correlation coefficient between simulative mean chromatograms.

## 2 Experimental design, materials and methods

### 2.1 Extraction of essential oils

All *J. rigida* needles were air-dried and powdered, and were stored in the dark at −20 °C for further analysis. The essential oils of *J. rigida* was isolated by supercritical CO_2_ fluid extraction technology used by Meng et al. [2]. The optimum condition is at a pressure of 18 MPa and a temperature of 40 °C and an extraction time of 120 min. The essential oil was stored in tightly closed dark vials and covered with aluminum foil at 4 °C until further analysis. The essential oil was obtained as a light yellow liquid and had specific aroma.

### 2.2 Principal component analysis (PCA)

Principal component analysis (PCA) was performed using SPSS software (SPSS for Windows 19.0, SPSS Inc., USA) for the chemometrics of essential oils [3]. Differences in chromatograms of samples mainly existed due to variations in the common peaks. To evaluate the discrimination capacity of the common constituents, PCA was conducted using the RPAs of common peaks using HCA input data. The first two principal components contained the most information of all variables, accounting more than 85% of total variability. The score plot of the first three principal components, PC1 and PC2, visually revealed a positive influence on quality evaluation of *J. rigida* from different regions.
